# Trans-generational desensitization and within-generational resensitization of a sucrose-best neuron in the polyphagous herbivore *Helicoverpa armigera* (Lepidoptera: Noctuidae)

**DOI:** 10.1038/srep39358

**Published:** 2016-12-14

**Authors:** Ying Ma, Jingjing Li, Qingbo Tang, Xuening Zhang, Xincheng Zhao, Fengming Yan, Joop J. A. van Loon

**Affiliations:** 1 The Institute of Chemical Ecology, Henan Agricultural University, Zhengzhou, 450002, China; 2 Zhengzhou City River Management Office, Zhengzhou, 450006, China; 3 Laboratory of Entomology, Wageningen University, Droevendaalsesteeg 1, 6708 PB Wageningen, The Netherlands

## Abstract

Dietary exposure of insects to a feeding deterrent substance for hours to days can induce habituation and concomitant desensitization of the response of peripheral gustatory neurons to such a substance. In the present study, larvae of the herbivore *Helicoverpa armigera* were fed on diets containing either a high, medium or low concentration of sucrose, a major feeding stimulant. The responsiveness of the sucrose-best neuron in the lateral sensilla styloconica on the galea was quantified. Results showed the response of the sucrose-best neuron exposed to high-sucrose diets decreased gradually over successive generations, resulting in complete desensitization in the 5^th^ and subsequent generations. However, the sensitivity was completely restored in the ninth generation after neonate larvae were exposed to low-sucrose diet. These findings demonstrate phenotypic plasticity and exclude inadvertent artificial selection for low sensitivity to sucrose. No significant changes were found in the sensitivity of caterpillars which experienced low- or medium-sucrose diets over the same generations. Such desensitization versus re-sensitization did not generalise to the phagosimulant *myo*-inositol-sensitive neuron or the feeding deterrent-sensitive neuron. Our results demonstrate that under conditions of high sucrose availability trans-generational desensitization of a neuron sensitive to this feeding stimulant becomes more pronounced whereas re-sensitization occurs within one generation.

All animals have chemosensory receptor neurons that respond to phagostimulant compounds and potentially bitter tastants in foods^1^,^2^,^3^,^4^,^5^,^6^. The importance of food chemistry and chemosensory detection in the evolution and maintenance of animal-food associations has been well documented for rats^7^,^8^, mice^9^,^10^,^11^, *Drosophila*^12^, lepidopteran insects^13^,^14^,^15^,^16^, and other insect species^17^,^18^,^19^,^20^. An important issue is to understand the evolutionary role of phenotypic plasticity of sensory systems elicited by changing environmental factors, for example food availability and nutritional quality^9^,^21^,^22^,^23^,^24^. In particular, a period of exposure to specific compounds can profoundly alter subsequent responsiveness to chemical stimuli^15^,^25^,^26^. In most investigations, changes in sensitivity develop over a period of hours to days^27^,^28^,^29^. Little is known, however, about the effects of trans-generational exposure on chemosensory sensitivity. Here, we investigate the trans-generational exposure to diets differing in sugar content on the responsiveness of gustatory receptor neurons in a polyphagous insect herbivore.

It has been well documented that food discrimination of lepidopteran caterpillars is governed by the activity of gustatory neurons in the medial and lateral sensilla styloconica on the maxillary galea to stimulants and deterrents in host plants^30^. It is well established that the sensitivity of these gustatory neurons can be modified by dietary experience that also affected food selection behaviour^13^,^31^. In most cases, after a period of exposure to diets containing a chemical stimulus, in later larval instars the gustatory neurons exhibited reduced sensitivity to the stimulus^27^,^28^,^29^,^32^,^33^,^34^.

The process of gustatory desensitization of sensilla styloconica to feeding deterrent substances depends on the insect species and the deterrent itself. Some studies documented that the sensitivity of the deterrent neuron in the sensilla styloconica decreased significantly but not completely after a chronic dietary exposure to the deterrent substance from neonates to the final instar^27^,^28^. In other cases, several days of dietary exposure to a deterrent resulted in the complete desensitization of the deterrent neuron^29^,^34^,^35^. Such desensitization of peripheral gustatory neurons to feeding deterrent substances could be mediated by gustatory transduction pathways, centrifugal control by the central gustatory system or post-ingestive mechanisms^21^,^29^,^36^.

It is known that the maxillary sensilla styloconica of caterpillars of all lepidopteran species studied contain a “sucrose-best”neuron, the activation of which leads to stimulation of feeding behavior^37^,^38^,^39^,^40^,^41^,^42^. Compared to the number of reports on the plasticity of gustatory neurons in response to exposure to feeding deterrents, the effects of exposure to varying levels of phagostimulants on the gustatory sensitivity of plant-feeding insects have received little attention. After exposure for a few hours to food containing a high level of carbohydrates but a low level of protein, the responsiveness to sucrose in the blowfly *Phormia regina*, and the caterpillars of the lepidopterans *Spodoptera littoralis* and *Grammia geneura* exhibited desensitization to sucrose^38^,^43^,^44^. Little is known, however, about desensitization and resensitization of sucrose-best neurons in sensilla styloconica of caterpillars elicited by varying levels of dietary sucrose.

The cotton bollworm, *Helicoverpa armigera* (Hübner) (Lepidoptera: Noctuidae), is a typical polyphagous species feeding on at least 160 plant species^45^,^46^,^47^. Taste neurons sensitive to sucrose and the deterrent azadirachtin are located in the lateral sensillum, whereas neurons responding to the sugar alcohol *myo*-inositol and the deterrents strychnine and strophanthin-K reside in the medial sensillum^27^,^37^,^48^,^49^,^50^. Deterrent-sensitive neurons in the maxillary sensilla of *H. armigera* caterpillars reared on artificial diets containing either strychnine or strophanthin-K from neonate to the 5^th^ instar exhibited reduced sensitivity to the two chemicals compared with the caterpillars reared on normal diets^27^. Sinigrin was found to be deterrent to *H. armigera* caterpillars in dual-choice leaf disk assays and it excites the same deterrent neuron in the medial sensillum as strychnine and strophantin-K. (Tang *et al*., unpubl. results). In the present study, the electrophysiological activity of the sucrose neuron in the lateral sensillum of *H. armigera* larvae that experienced diets differing in the content of the major feeding stimulant sucrose, high-sucrose (HS), medium-sucrose (MS) and low-sucrose (LS) diets were investigated.

We addressed the following inter-related questions: (1) Can chronic exposure to HS diets during larval development induce desensitization of the sucrose-best neuron in the lateral sensillum styloconicum of *H. armigera*? (2) Can the chronic exposure to HS diets result in a complete desensitization of the sucrose-best neuron? If so, how long is needed to achieve this? (3) Can the sensitivity of the sucrose-best neuron be fully restored upon exposure to a normal dietary sucrose content and how long is needed for full restoration? (4) Does exposure of caterpillars to LS diets induce plasticity of the sucrose-best neuron?

## Results

**Figure 1:**
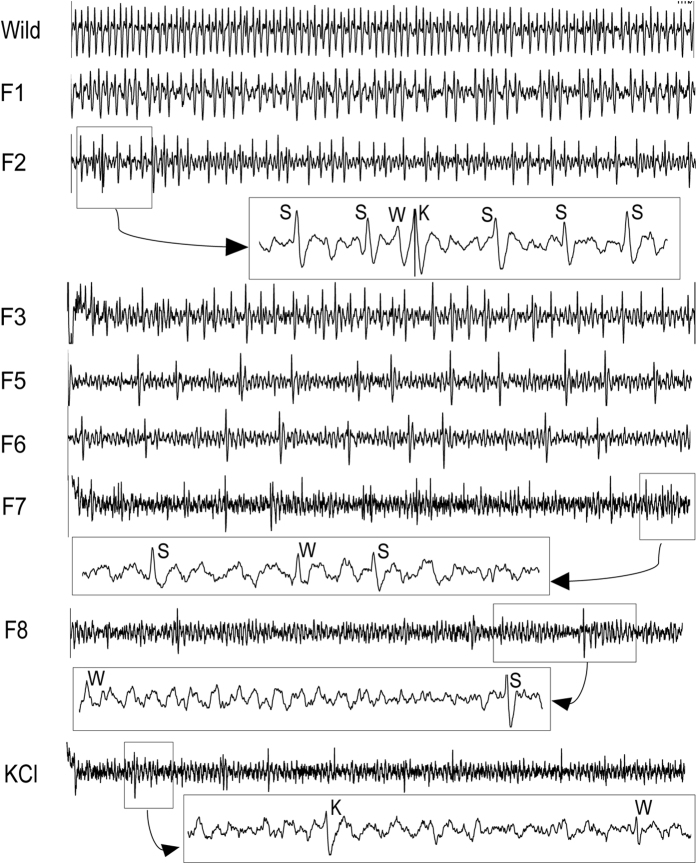
Representative recordings of electrophysiological activity of the gustatory receptor neurons in the lateral sensillum of larvae exposed to high-sucrose (HS) diet in successive generations. Traces represent electrophysiological responses to 1 mM sucrose. Sucrose was dissolved in 2 mM KCl. The duration of each trace is 1 s (except those in rectangles). Spikes in each big rectangle show the expansions of part of the corresponding trace (the small rectangle), suggesting that different gustatory receptor neurons were activated. K: the “largest-sized”spikes from the salt responding neuron. S: the medium-sized spikes from the “sucrose-best” neuron; W: the small-sized spikes from the “water” responding neuron; Note that the medium-sized spikes from the sucrose-best neuron had the dominating activities in most traces.

With the aid of Autospike software plus visual inspection, spikes in the responses from lateral sensilla of the 5^th^ instar caterpillars of *H. armigera* with different feeding experiences were sorted out and assigned to different types of gustatory receptor neurons known to be present in each chemosensillum. By comparing to the responses to sucrose with those obtained to KCl, at least three types receptor neurons were identified as the “salt”, “sucrose” and “water” best responding unites (see exemplary traces in Fig. 1). In most traces obtained from the responses to sucrose, the dominating “medium-sized” spikes were from the sucrose-best neuron, the “largest-sized” spikes were from the “salt” best responding neuron, and few “small-sized” spikes were from the “water” best responding neurons (see exemplary traces in Fig. 1).

### Desensitization to sucrose after HS-exposure over generations

**Figure 2:**
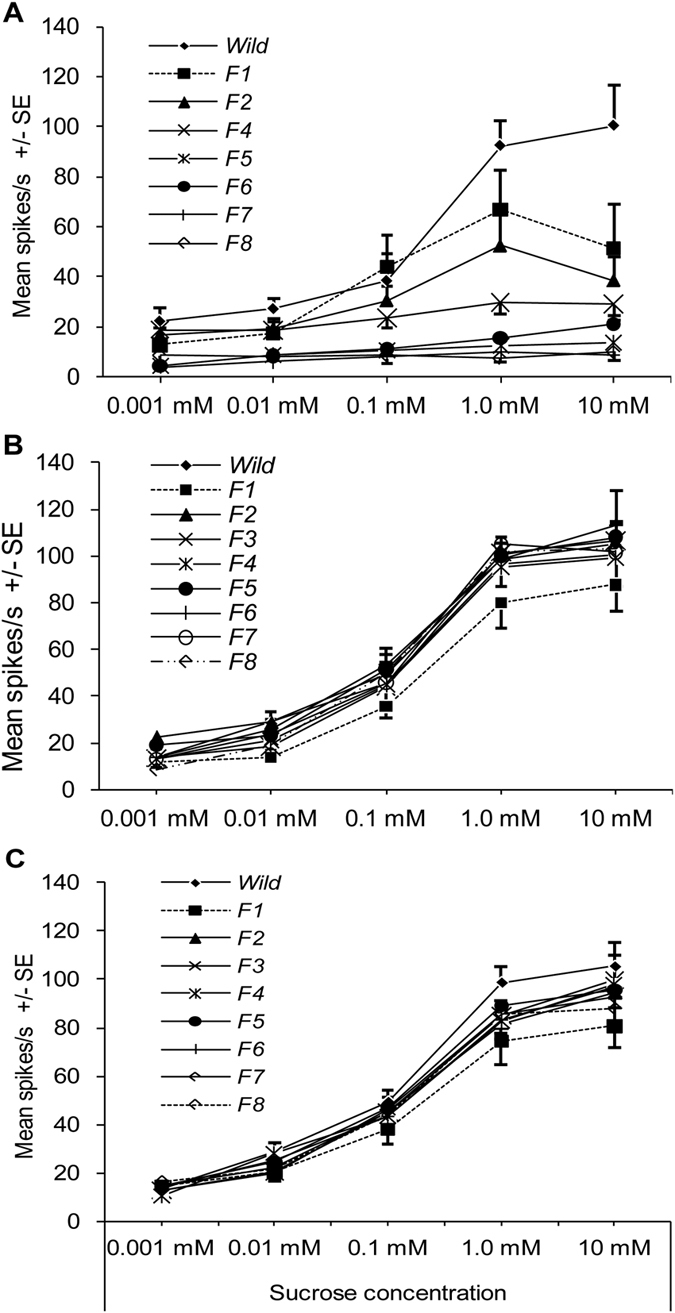
Dose–response curves of electrophysiological activity recorded from the sucrose-best neuron in the lateral sensillum of caterpillars reared on diets containing different sugar levels over successive generations. Each point represents the mean response frequency +/− SE of the sucrose-best neuron of 5^th^ instar *H. armigera* caterpillars in different generations (F1 to F8) in response to a concentration series of sucrose. (**A**) Reared on HS diet (high-sucrose artificial diet); (**B**) reared on LS diet (low-sucrose artificial diet); (**C**) reared on MS diet (medium-sucrose artificial diet). The tested number of caterpillars exposure to HS diet in each generation from *Wild*, F1, F2, F4, F6, F7 and F8 were 22, 20, 24, 20, 20, 20 and 20. And the tested number of caterpillars exposure to LS diet in each generation from *Wild*, F1 to F8 were 22, 12, 12, 15, 11, 13, 10, 18 16, respectively. The tested number of caterpillars exposure to MS diet in different generations from *Wild*, F1 to F8 were 14, 12, 14, 12, 13, 12, 13, 12 and 14 respectively.

The sensitivity of the sucrose-best neuron in the lateral sensillum of *H. armigera* caterpillars exposed to HS diet was significantly affected by both generation and sucrose concentration (univariate ANOVA: generation, df = 7, F = 42.707, *P* < 0.001; sucrose concentration, df = 4, F = 20.598, *P* < 0.001). There was also a significant interaction between generation and sucrose concentration affecting response frequency (univariate ANOVA: generation × concentration, df = 28, F = 3.066, *P* < 0.001). The 5^th^ instar caterpillars exposed to HS diet exhibited decreased sensitivity of the sucrose-best neuron in the lateral sensillum of ***Wild*** caterpillars (see exemplary trace “***F1***” in Fig. 1) (Fig. 2A). In subsequent generations sensitivity to sucrose decreased graduallyto a slight response in F5 generation. The response frequency of the sucrose-best neuron to sucrose from F5 to F8 generation were not significantly different from those to the control solvent KCl (Post-hoc SNK-test after univariate ANOVA: all *P* = 0.143) (see exemplary traces in Fig. 1) (Fig. 2A).

The sucrose-best neuron in ***Wild*** caterpillars of *H. armigera* displayed obvious dose-dependent responses to a concentration series of sucrose from 0.001 mM to 10 mM (***Wild***in Fig. 2A). Similarly, caterpillars exposure to HS diet in F1, F2 and F4 generations also exhibited dose-dependent response patterns to sucrose but the sensitivity was significantly lower than that in ***Wild***(Post-hoc SNK-test after univariate ANOVA: all *P* < 0.01) (***F1***,***F2***and***F4***in Fig. 2A). However, caterpillars exposed to HS diet from the 5^th^ generation to the 8^th^ generation did no longer display a dose-dependent response pattern to sucrose and the response intensity was significantly lower than that in the previous generations (Post-hoc SNK-test after univariate ANOVA: all *P* < 0.01) (Fig. 2A). So, it is apparent that the sucrose-best neuron of 5^th^ instar larvae had become completely desensitized in the 5^th^ generation since exposure to high-sucrose diets.

### Responsiveness to LS/MS dietary exposure over generations

**Figure 3:**
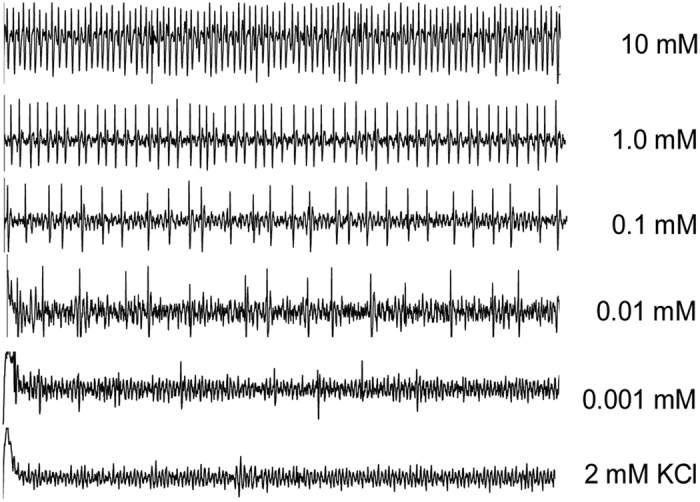
Representative recordings of electrophysiological activity obtained from the sucrose-best neuron in the lateral sensillum of caterpillars exposed to low-sucrose (LS) artificial diets in the generation *F1-LS*. Recordings in this figure originated from caterpillars of the F1 generation exposed to LS diet (*F1-LS*). Each concentration of sucrose was dissolved in 2 mM KCl. The duration of each trace was 1 s.

To establish that it was exposure to a HS diet across successive generations that produced the desensitization of the sucrose-best neuron, the responsiveness of the sucrose-best neuron in the lateral sensillum of *H. armigera* caterpillars exposed to either LS diet or MS diet over generations was also investigated (Fig. 2B,C). Firstly, the sucrose-best neuron of caterpillars exposed to LS diet displayed dose-dependent responses in each generation from ***Wild*** to ***F8*** (Fig. 2B and see exemplary traces in Fig. 3). The sensitivity of the sucrose-best neuron in caterpillars reared on LS diet was significantly affected by both generation and sucrose concentration (univariate ANOVA: generation, df = 8, F = 2.855, *P* = 0.004; concentration, df = 4, F = 325.953, *P* < 0.001; generation × concentration, df = 32, F = 0. 468, *P* = 0.995). Compared to ***Wild***, the response intensity of the sucrose-best neuron of caterpillars in ***F1-LS*** decreased significantly (Post-hoc SNK-test after univariate ANOVA: *P* < 0.01), whereas the sensitivity of the sucrose-best neuron in***F2-LS*** was significantly higher than that of ***F1-LS*** (Post-hoc SNK-test after univariate ANOVA: *P* < 0.05) and was similar to that of the ***Wild***and the **F3**~**F8** generations (Post-hoc SNK-test after univariate ANOVA: *P* = 0.136) (Fig. 2B).

Similarly, the responsiveness of the sucrose-best neuron in caterpillars exposed to MS diet also displayed dose-dependent responses from ***Wild*** to ***F8*** generation (Fig. 2C). Generation of exposure to MS-diet had no significant effect on the sensitivity of the sucrose-best neuron (univariate ANOVA: generation, df = 8, F = 1.644, *P* = 0.110; concentration, df = 4, F = 373.503, *P* < 0.001; generation × concentration, df = 32, F = 0.490, *P* = 0.992).

### Responsiveness of caterpillars reared on HS diet and switched to LS or MS diets

**Figure 4:**
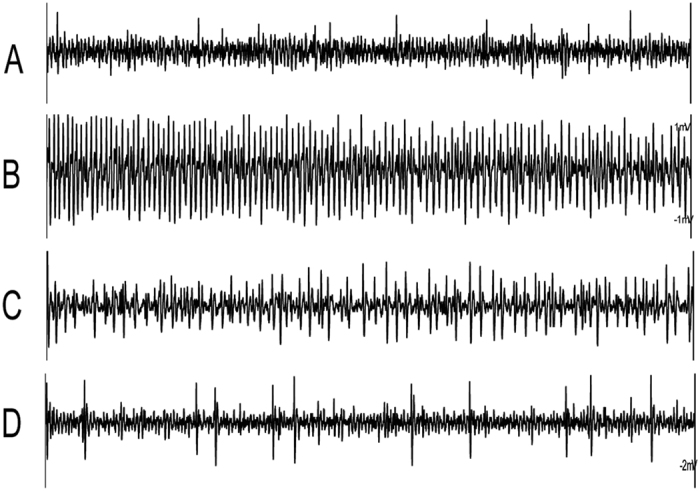
Representative recordings of electrophysiological activity of the sucrose-best neuron in the lateral sensillum to 1 mM sucrose in caterpillars exposed to different artificial diets. (**A**) Caterpillars exposed to HS diet over eight successive generations (*F8-HS*); (**B**) *F9-*caterpillars exposed to the LS diet (*F8-HS* + *F9-LS*); (**C**) *F9-*caterpillars exposed to the medium-sucrose artificial diet (*F8-HS* + *F9-MS*); (**D**) caterpillars exposed to HS diet over nine successive generations (*F9-HS*); Sucrose was dissolved in 2 mM KCl. The duration of each trace was 1 s.

**Figure 5:**
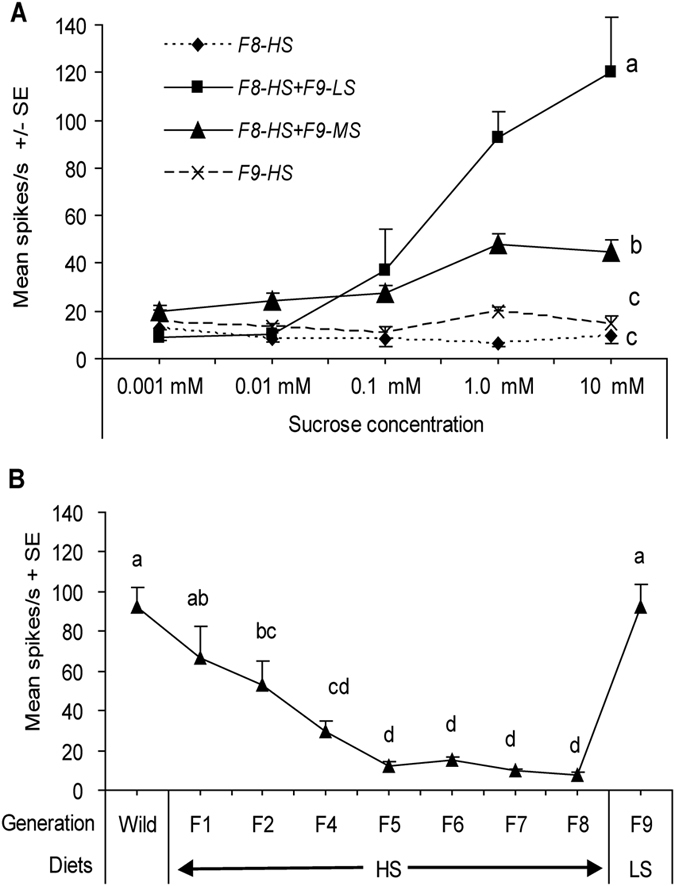
Dose–response curves recorded from the sucrose-best neuron in the lateral sensillum of caterpillars exposure to HS diet over eight generations and then switched to other diets. Each point represents the mean response frequency +/− SE of the sucrose-best neuron of 5^th^ instar *H. armigera* caterpillars in the response to a series of sucrose concentrations. (**A**) *F8-HS* (n = 20), *F8-HS *+* F9-LS* (n = 22), *F8-HS *+* F9-MS* (n = 24) and *F9-HS* (n = 20) caterpillars; (**B**) responses to 1 mM sucrose in *Wild* (n = 22), *F1-HS* (n = 20) to *F8-HS* (n = 20), and *F8-HS *+* F9-LS* (n = 22) caterpillars. Means with different low case letters are significantly different (Post-hoc SNK-test: *P* < 0.05).

The sensitivity of the sucrose-best neuron of caterpillars that experienced one diet over eight generations and then were switched to other diets in the ninth generation were tested to investigate whether the changing of diets could result in a taste change. Three groups of caterpillars experienced different artificial diets differing in sugar content were investigated as described in materials and methods. In Group I, the responsiveness of the sucrose-best neuron was significantly higher after caterpillars of *H. armigera* exposed to HS diet for eight generations were exposed to LS or MS diet (univariate ANOVA: experience, df = 3, F = 49.112, *P* < 0.001; concentration, df = 4, F = 23.262, *P* < 0.001; experience × concentration, df = 12, F = 12.742, *P* < 0.001) (see exemplary traces in Figs 4 and 5A). When neonate F9-caterpillars were exposed to LS diet (***F8-HS***** + *****F9-LS***), the response of the sucrose-best neuron of 5^th^ instar caterpillars was significantly higher than that of ***F8-HS**, **F8-HS***** + *****F9-MS*** and***F9-HS***(Post-hoc SNK-test after univariate ANOVA: all *P* < 0.01) (Fig. 5A) and similar to that of ***Wild*** and ***F1-HS***(Post-Hoc SNK Test after ANOVA: *P* = 0.075) (Fig. 5B).

Similar to ***F8-HS***** + *****F9-LS***, F9-neonate caterpillars derived from ***F8-HS*** exposed to MS diet (***F8-HS***** + *****F9-MS***) displayed higher responsiveness of the sucrose-best neuron in the 5^th^ instra than ***F8-HS*** 5^th^ instar caterpillars (Post-hoc SNK-test after univariate ANOVA: *P* < 0.01), but responsiveness was significantly lower than that of ***F8-HS***** + *****F9-LS*** caterpillars (Post-hoc SNK-test after univariate ANOVA: *P* < 0.01) (Fig. 5A).

### Responsiveness of caterpillars reared on LS/MS diet and then switched to other diets

**Figure 6:**
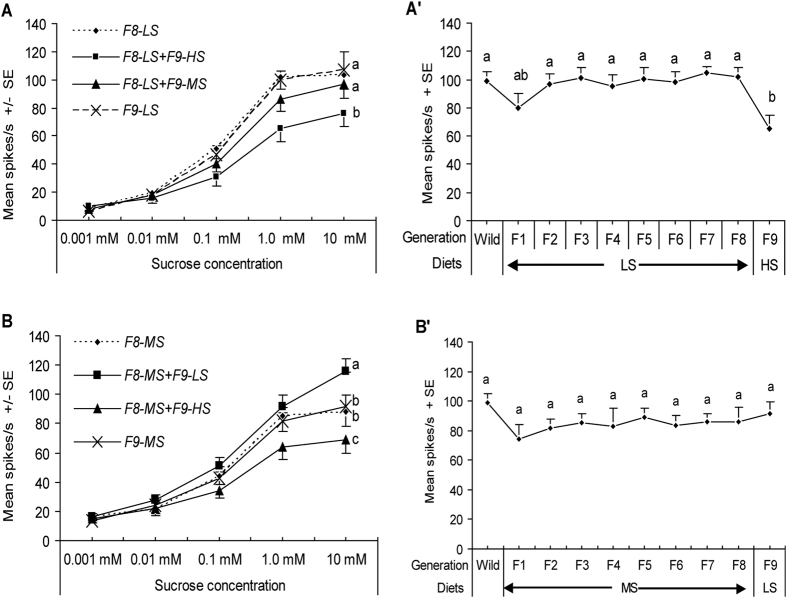
Dose–response curves recorded from the sucrose-best neuron in the lateral sensilla of caterpillars exposed to LS or MS diet over eight generations and then switched to other diets. Each point represents the mean response frequency +/− SE of the sucrose-best neuron of 5^th^ instar caterpillars. (**A**) Responsiveness of caterpillars exposed to LS diet over eight generations (*F8-LS*, n = 16) and switched to MS (*F8-LS *+* F9-MS*, n = 12) or HS diets (*F8-LS *+* F9-HS*, n = 14; *F9-LS*, n = 10); (A′) Response curve to 1 mM sucrose in caterpillars exposed to LS diet over eight generations (replicates of caterpillars were the same as in Fig. 2B) and then exposed to HS diet (*F8-LS* + *F9-HS*, n = 14). (**B**) Caterpillars exposed to MS diet over eight generations (*F8-MS*, n = 14) and switched to HS/LS diets (*F8-MS *+* F9-LS*, n = 14; *F8-MS *+* F9-HS*, n = 13; *F9-MS*, n = 12); (B′) Response curve to 1 mM sucrose in caterpillars exposed to MS diet over eight generations (replicates of caterpillars were the same as in Fig. 2C) and then exposed to LS diet (*F8-MS* + *F9-LS*, n = 14). Different letters represent significant difference of the mean response frequency in different generations (Post-hoc SNK-test: *P* < 0.05).

In Group II, after the F9-neonate caterpillars derived from generation ***F8-LS*** were exposed to HS diet or MS diet, the responsiveness of the sucrose-best neuron of the 5^th^ instar caterpillars in ***F8-LS*** **+** ***F9-MS*** decreased but not significantly (Post-hoc SNK-test after univariate ANOVA: *P* = 0.292), while that of ***F8-LS***** +** ***F9-HS***caterpillars decreased significantly compared to that of ***F8-LS***and***F9-LS*** caterpillars (Post-hoc SNK-test after univariate ANOVA: all *P* < 0.05) (Fig. 6A). For example, 1 mM sucrose elicited 65.1 ± 9.34 spk s^−1^ in ***F8-LS***** + *****F9-HS***caterpillars, which was significantly lower than 101.9 ± 8.66 spk s^−1^ recorded from ***F8-LS*** and ***F2-LS*** to ***F7-LS*** caterpillars (Post-Hoc SNK Tests after ANOVA: all *P* < 0.05) (Fig. 6A′).

Similarly, in Group III after F9-neonate caterpillars derived from ***F8-MS*** were exposed to LS diet (***F8-MS***** + *****F9-LS***) or HS diet (***F8-MS***** + *****F9-HS***), the responsiveness of the sucrose-best neuron also differed (univariate ANOVA: experience, df = 3, F = 8.773, *P* < 0.001; concentration, df = 4, F = 162.671, *P* < 0.001; experience × concentration, df = 12, F = 1.136, *P* = 0.355). Firstly, compared to that in ***F8-MS*** and ***F9-MS***caterpillars, the sensitivity of the sucrose-best neuron of ***F8-MS***** + *****F9-LS*** 5^th^ instar caterpillars was significantly increased (Post-hoc SNK-test after univariate ANOVA: all *P* < 0.05) (Fig. 6B). On the contrary, the sucrose-best neuron of ***F8-MS***** + *****F9-HS*** caterpillars had significantly lower sensitivity compared to that of ***F8-MS*** and ***F9-MS*** caterpillars (Post-hoc SNK-test after univariate ANOVA: all *P* < 0.05) (Fig. 6B). However, 1 mM sucrose elicited similar response intensity in the sucrose-best neuron of ***Wild**, **F1-MS*** to ***F8-MS***, and ***F8-MS***** + *****F9-LS***caterpillars (Post-Hoc SNK Test after ANOVA: *P* = 0.445) (Fig. 6B′).

### Effects of diets with different sucrose levels on the deterrent and myo-inositol neurons

**Figure 7:**
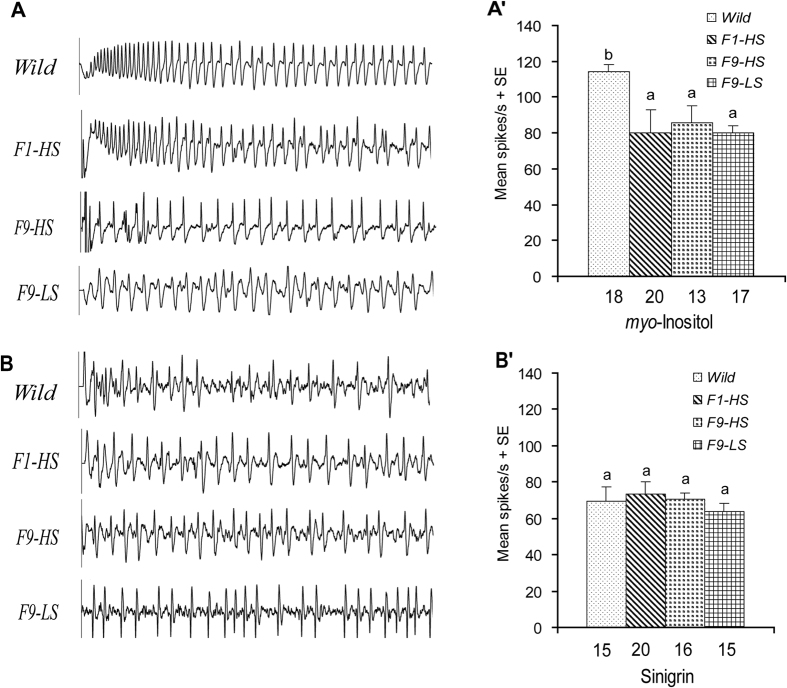
Representative recordings of electrophysiological activity and comparison of response intensity of gustatory neurons in the medial sensillum to *myo*-inositol and sinigrin from caterpillars exposed to diets differing in sugar level. (**A**) representative recording from neuron to myo-inositol (300 ms); (A′) comparison of response intensity from neuron to myo-inositol; (**B**) Representative recording from neuron to sinigrin (300 ms); (B′) comparison of response intensity from neuron to sinigrin. Columns represent the mean response frequency +/− SE in the response to 1 mM *myo*-inositol (A′) and to 1 mM sinigrin (B′). *Numbers* below the columns show the numbers of insects tested. SNK post hoc test for one-way ANOVA was used to compare mean spike frequencies of the sensilla across caterpillars with different feeding experiences (*P* < 0.05). Means with different lower case letters are differ significantly (*P* < 0.05).

The effects of HS diet or LS diet on the responsiveness of the *myo*-inositol-sensitive neuron and the sinigrin-sensitive neuron in the medial sensillum of *H. armigera* caterpillars were investigated. Results showed that the sensitivity of the *myo*-inositol-sensitive neuron in the medial sensillum of caterpillars in ***Wild*** was significantly higher than that of caterpillars in ***F1-HS**, **F9-HS*** and ***F9-LS***in the response to 1 mM *myo*-inositol (Post-Hoc SNK Test after ANOVA: *P* < 0.05), while the response frequencies among the latter three groups were similar (Post-Hoc SNK Test, *P* = 0.840) (Fig. 7A,A′). On the other hand, 1 mM sinigrin elicited a similar response from the deterrent neuron in the medial sensillum of caterpillars from ***Wild**, **F1-HS**, **F9-HS*** and ***F9-LS*** (Post-Hoc SNK Test after ANOVA: *P* = 0.915) (Fig. 7B,B′).

## Discussion

Sugars serve as universal sources of metabolic energy to organisms and most animals have the ability to taste sugars that in many species constitute primary stimulatory signals for feeding^51^. The sugar concentrations investigated here are within the range of reported levels in different plants and plant organs on which *H. armigera* is known to feed. The sugar content varies greatly in the principal host plants of the polyphagous herbivore *H. armigera, e.g.* 1.73~5.37% of dry weight in the green leaves of tobacco, *ca.* 8% in tomato fruits in green ripening stage, 16% in corn stalks, and up to 20.5% in floral buds of cotton^52^,^53^,^54^,^55^,^56^,^57^. We found that dietary exposure to the HS diet desensitized the sucrose-best gustatory neuron. The desensitization developed to stronger degrees over successive generations, reaching its maximum after five generations of exposure and remaining at this level in the next three generations. Once the caterpillars were exposed to the LS diet, the sucrose-best neuron was completely restored to its wildtype condition, showing that no selection for low sensitivity had occurred after eight generations. Taste plasticity induced by early exposure to stimuli within one generation was also reported in other insect species and animals. For example, honeybees *Apis mellifera* decreased the gustatory responsiveness to sucrose after switching to an sucrose-enriched diet for 6 h to 24 h^18^, and adult rats exposure to a sodium-deficient diet for 10 days selectively decreased NaCl responses in the chorda tympani nerve^26^. Mice that experienced unlimited exposure to sucrose diets in early life reduces motivation to acquire sucrose in adulthood^58^, but no similar findings have yet been reported on trans-generational taste plasticity in insects and other animals except Dias and Ressler reported that the fear odor could be inherited transgenerationally at behavioral, neuroanatomical and epigenetic levels from parental olfactory experiences via parental gametes^59^.

The exposure of caterpillars to HS diet for successive generations resulted in a gradual desensitization of the sucrose-best neuron, while the exposure to either LS diet or MS diet for successive generations failed to elicit significant change in the sensitivity of the sucrose-best neuron of *H. armigera*. On the other hand, after the neonate caterpillars exposed to the same diet for eight generations were exposed to another diet, the sensitivity of the sucrose-best neuron in the 5^th^ instar caterpillars demonstrated significant plasticity in most situations. For example, the exposure to LS diet elicited stronger responses of ***F8-HS *****+***** F9-LS*** or ***F8-MS *****+***** F9-LS*** caterpillars compared to that of ***F8-HS*** or in ***F8-MS***caterpillars (Figs 5A and 6B), while the exposure to HS diet desensitized the sucrose-best neuron of caterpillars in ***F8-LS *****+***** F9-HS*** or in ***F8-MS *****+***** F9-HS***compared to that in ***F8-LS*** or in ***F8-MS***(Fig. 6A,B). This plasticity of the sucrose-best neuron of *H. armigera* caterpillars was at least in part consistent with plasticity in the chemosensitivity to sucrose in the locust *Locusta migratoria*, the blowfly *P. regina*, and the caterpillars *S. littoralis* and *G. geneura* exposed to high-sucrose/low-protein diets or to low-sucrose/high-protein diets^38^,^43^,^44^,^60^.

The current findings also show that different sucrose contents in artificial diets elicited different degrees of desensitization or resensitization of the sucrose-best neuron in *H. armigera*. For example, LS diet elicited a stronger response in caterpillars of ***F8-HS *****+***** F9-LS*** than the response elicited by MS diet in ***F8-HS *****+***** F9-MS*** (Fig. 5A). On the contrary, the HS diet significantly desensitized the sucrose-best neuron of caterpillars in ***F8-HS *****+***** F9-HS**(**F9-HS***) compared to the response of caterpillars in ***F8-HS *****+***** F9-MS*** elicited by MS diet (Fig. 5A). Similarly, HS diet elicited a significantly lower response in the caterpillars of ***F8-LS *****+***** F9-HS*** than MS did in ***F8-LS *****+***** F9-MS*** (Fig. 6A). Similar findings on the plasticity of the sensitivity to sucrose were also found in *S. littoralis* exposed to diets differing in sucrose and protein content for hours^61^.

The shifts in carbohydrate/protein ratio in the HS/LS/MS artificial diet in the current experiment might have had effects on the development of *H. armigera* caterpillars as reported for *Heliothis virescens*^62^ and *Helicoverpa zea*^63^. However, we found that caterpillars of *H. armigera* exposed to HS and LS diets in our rearing colonies had similar pupal development time, pupal mass, and pupal survival (% eclosing) as well as larval development time, while larval survival (% pupating) and the number of eggs produced by ***F9-HS*** individuals (larval survival: 69.53%; number of eggs/female: 344.84) was lower than those of the ***F9-LS*** individuals (larval survival: 85.41%; number of eggs/female: 460.47) and the ***F9-MS*** individuals (larval survival: 93.26%; number of eggs/female: 622.83). On the contrary, ***F9-MS*** individuals had higher pupal survival (% eclosing) (92.08%) than that of ***F9-HS*** individuals (74.55%) as well as ***F9-LS*** individuals (84.10%). The current data suggest that the plasticity of the sucrose-best neuron in *H. armigera* depends on dietary sucrose content rather than on protein/sucrose ratio or altered development. We consider this explanation plausible for two reasons. First, the absolute content of protein in LS, HS and MS diets was similar and the protein/carbohydrate ratio in the current study was not so unbalanced as that in the studies on *H. virescens, H. zea* and *G. geneura*^38^,^62^,^63^. Bernays *et al*.^38^ regarded the sensitivity changes of taste neurons to sucrose and amino acids in *G. geneura* to reflect carbohydrate but not protein imbalance. Second, the HS diet or the LS artificial diet in the current experiment did decrease the sensitivity of the neuron sensitive to *myo*-inositol in F1 generation, but did not change further on the basis of sugar concentration or trans-generationally, indicating artificial diets differing in sugar concentrations and generational exposure had no bearing on overall plasticity. Third, the sensitivity of the deterrent neuron to sinigrin in *H. armigera* did not exhibit any plasticity to any sugary diets in any tested generations, suggesting that there was no general physiological effect on *H. armigera* caterpillars experiencing LS diet or HS diets for eight generations.

It is known that the *myo*-inositol serves as a phagostimulant and sinigrin as a deterrent to non-adapted lepidopteran caterpillars^64^,^65^,^66^. Our data demonstrated that neither exposure of F1 caterpillars nor trans-generational exposure to HS/LS diets desensitized the sinigrin-sensitive neuron in the medial sensillum of *H. armigera*. On the other hand, the sensitivity of the *myo*-inositol-sensitive neuron in the medial sensillum was lower in ***F1-HS*** caterpillars compared to that in ***Wild***caterpillars, but did not exhibit significant plasticity in successive generations regardless of the diets experienced. We interprete this as evidence for specificity of desensitization of the sucrose-best neuron caused by exposure to the HS diet across successive generations. Specificity in the desensitization of gustatory neurons was also reported for other caterpillars, *e.g.* a gustatory neuron in the medial sensillum of caterpillars of *G. geneura* exposed to a high-sucrose diet was less responsive to sucrose while the response to fructose in the lateral sensillum was constant^38^. Similarly, dietary exposure to caffeine in *Manduca sexta* caterpillars desensitized a caffein-sensitive neuron but neither the sucrose neuron nor the *myo*-inositol-sensitive neuron were affected^29^.

It was unexpected that not only the sensitivity of the sucrose-best neuron in ***F1-HS*** caterpillars decreased compared to that in ***Wild*** caterpillars but also that of ***F1-LS*** and ***F1-MS*** caterpillars. Decreased sensitivity was also observed in the *myo*-inositol-sensitive neuron of caterpillars in generation ***F1-HS***. We postulate that ***F1***caterpillars had not yet completely adapted to the artificial rearing diet but ***F2***caterpillars had, as judged by the recovered sensitivity of the sucrose-best neuron in ***F2-LS***(Fig. 2B) or ***F2-MS***caterpillars (Fig. 2C). Another sign of ongoing adaptation was the observation that 4^th^ or the 5^th^ instar caterpillars of *H. armigera* captured directly from the natural fields had a higher mortality than that of second or third instar caterpillars if reared on normal artificial diets (unpublished data).

Desensitization of the deterrent-sensitive neurons in *H. armigera* and other caterpillar species in general took 2 h to 24 h, however, it was not complete^27^,^28^,^34^,^36^. Our results show that it took five generations of exposure to a high dietary sucrose concentration to completely desensitize the sucrose-best neuron. Transgenerational experiments on desensitization to deterrents have not been performed to our knowledge. Several mechanisms may explain exposure-elicited desensitization, *e.g.* a reduced number of membrane-bound gustatory receptor proteins^10^,^67^,^68^,^69^ and/or down-regulated secondary messenger signal-transduction pathways^70^,^71^,^72^. The sensitivity of the sucrose-best neuron in the last instar caterpillars of *H. armigera* declined gradually over successive generations, suggesting that the reduced sensitivity could be transferred from one generation to the next and may involve an epigenetic phenomenon. Based on the full recovery of electrophysiological sensitivity within one larval generation, genetic selection on low sensitivity to sucrose can be excluded.

The regulation of gustatory plasticity in insects is clearly very complex. Notable in these responses is the observation that, after exposure to HS diet over generations, caterpillars of *H. armigera* showed lower responsiveness to sucrose, while after switching after eight generations to a LS diet higher responsiveness to sucrose was found. It seems likely that this restoration of the sensitivity of the sucrose-best neuron is driven, at least in part, by low a carbohydrate level in the haemolymph. Therefore, we consider the operation of a post-ingestive centrifugal nutritional feedback mechanism involving the central nervous system as the most parsimonious explanation for the sucrose exposure-elicited gustatory de- and resensitization. The exposure to a high dietary level of sucrose may result in a reduced sensitivity of the sucrose-best neuron in *H. armigera* since abundant dietary sucrose may not require gustatory sensitivity to sucrose and may save investments in maintaining sucrose chemoreception. Moreover, it was reported that the biogenic amines, c.a. the octopamine and serotonin in extracellular sensillum lymph, could adjust the sensitivity of olfactory receptor neurons of *M. sexta* by modulating the transepithelial potential of the accessory cells in the sensillum^73^. Therefore, it is possible to explore whether such neurotransmitters or hormone in extracellular sensillum a affected by artificial diets, resulting in direct or indirect plasticity of the sucrose-best neuron.

A nutritional feedback mechanism has been shown to operate within 5 min in response to injection of a mixture of amino acids into the haemocoel in the orthopteran *L. migratoria* and resulted in a reduced response of taste receptors to several amino acids^74^. Similarly, the gustatory response of labellar taste sensilla in the blowfly *P. regina* to glucose decreased significantly two hours after the injection of 1 M trehalose into the haemolymph^75^. Therefore, we hypothesize that the plasticity of the sucrose-best neuron in caterpillars of *H. armigera* induced by diets differing in sucrose content is related to variations in the levels of sugars in the haemolymph. In a next study, the levels of sugars in the haemolymph of *H. armigera* caterpillars after exposure to different levels of sucrose in the artificial diet will be quantified to test this hypothesis.

## Materials and Methods

### Insects

*Helicoverpa armigera* larvae were collected from a tomato field in Zhengzhou, Henan province, China, and were divided into different experimental groups. Based on the diet composition described by Wu & Gong^76^, three diets differing in sucrose concentration, high-sucrose (HS), medium-sucrose (MS) and low-sucrose (LS), were prepared to rear the experimental larvae. Including the sucrose in wheat bran, soybean powder and other dietary contents, the percentages of sucrose in the HS, MS and LS diets were 17.15%, 5.69% and 2.65% of dry weight, respectively which were within the range of sugar contents reported for natural host plants of *H. armigera* caterpillars (see Discussion). All colonies of *H. armigera* were maintained in the laboratory under controlled photoperiod (L16:D8) and temperature (27 ± 1 °C). Adults were supplied with a 10% v/v solution of sucrose in water.

**Figure 8:**
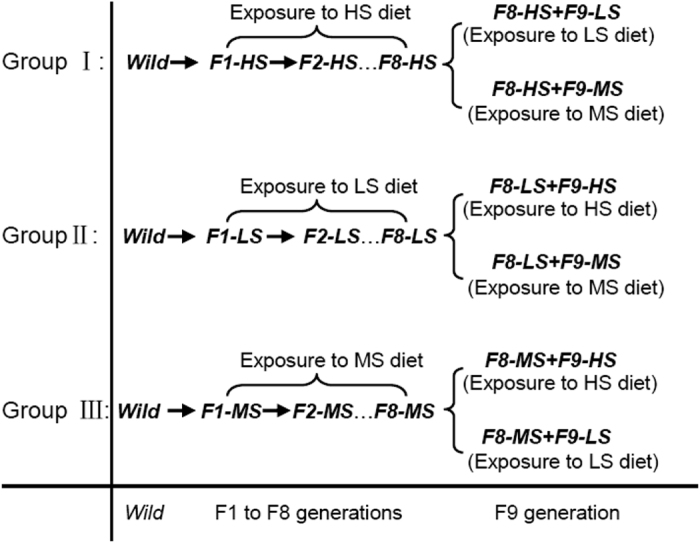
Timelines of exposure regimes and diet-switching of three groups of caterpillars exposed to diets differing in sucrose levels used in the experiments. HS diet: high-sucrose diet; LS diet: low-sucrose diet; MS diet: medium-sucrose diet.

Three groups of *H. armigera* caterpillars exposed to the three dietary sucrose levels were used in this experiment. Every group consisted of caterpillars captured directly from the field (***Wild***) as described above, caterpillars exposed to each diet for eight successive generations and caterpillars that were switched to the other two diets in the ninth generation as shown in Fig. 8.

### Chemicals

Sucrose, *myo*-inositol and sinigrin were obtained from Sigma Chemical Co. (purity > 99.5%). For electrophysiological tests, sucrose was presented in a series of concentrations from 0.001 to 10 mM dissolved in 2 mM KCl, an appropriate electrolyte for the electrophysiology of *Helicoverpa* caterpillars^27^,^77^. As a control for the specificity of effects of diets on the sensitivity of the sucrose-best neuron, we also tested whether diets containing different contents of sucrose have effects on the sensitivity of another phagostimulant neuron and a deterrent neuron, *i.e.* the independent phagostimulant neuron to *myo*-inositol and the deterrent neuron to sinigrin in the medial sensillum of *H. armigera* caterpillars^37^. Concentration of both chemicals was 1 mM, a concentration often used in lepidopteran taste studies^64^,^78^.

### Electrophysiological recording

The tip recording technique^79^,^80^ was used to investigate the electrophysiological activity of neurons in the lateral sensilla styloconica on the galea of *H. armigera* larvae. In brief, 5^th^ instar larvae between 24 h and 36 h (during the photophase) since the penultimate moult were transected between the first and second pair of thoracic legs and the excised head capsule was mounted on a silver wire electrode that was connected to the input of a pre-amplifier (Syntech Taste Probe DTP-1, Hilversum, The Netherlands). The maxillary lateral sensilla styloconica were investigated for the sensitivity to sucrose of caterpillars that had fed on diets differing in sucrose content, while the medial sensilla were stimulated to assess the sensitivity of the *myo*-inositol-sensitive neuron or the deterrent neuron. Recordings of electrophysiological activity were obtained from the sensillum styloconicum of one side of the mouthparts of each caterpillar to different concentrations of sucrose or *myo*-inositol or sinigrin using a glass microelectrode (tip diameter *ca.* 30 μm) filled with stimulus solution. Each caterpillar was only tested by one of the three stimuli with series concentration and then discarded. The order in which the series of sucrose concentrations were applied to a single sensillum was from low to high concentration of sucrose. The duration of each stimulation was 10 s and an interval of at least 3 min between two stimulations was observed. At least 10 larvae in each dietary group were tested for their sensitivity of a gustatory receptor neuron to the sucrose concentration series, *myo*-inositol or sinigrin. Amplified signals were digitized by an A/D-interface (IDAC-4, Syntech) and sampled and stored on a personal computer. Electrophysiological responses were quantified by counting the number of spikes in the first second after the start of stimulation.

Spikes were analyzed and counted visually by the experimenter with the aid of Autospike version 3.7 software (Syntech), running the sub-routine “Spike Conversion”, by which all spikes in a recording can be classified according to their amplitude but also their waveform (shape). We then, in general, applied an amplitude threshold and the scale bar to select the target spike groups originating from the “sucrose-best neuron” or other neurons tested. In general, this medium-sized spike type but not the “largest” spike type was identified from the “sucrose-best” neuron because these spikes had two distinct features: (1) it always exhibited the most regular waveform; (2) it had an obvious phasic temporal response pattern. Applying these criteria, amplitude, uniform waveform and phasic temporal pattern resulted into the counts we reported. We then verified the counts arrived at by visual inspection and adjusted the count when the software seemed to have unduly skipped or included a waveform. Such adjustments were within 5% of the count reported by the Autospike software.

### Data analysis

The mean response frequency, i.e. the number of spikes fired by the sucrose-best neuron in the lateral sensillum in the first second (spk.s^−1^) was calculated. For statistical analysis, the original number of spikes per second in response to each stimulus was square-root transformed. The univariate ANOVA with Student-Newman-Keuls (SNK) Post-hoc test was used to compare: (1) the mean response frequency of the sucrose-best neuron in caterpillars exposed to one type of artificial diet over eight successive generations in response to different concentrations of sucrose; (2) the mean response frequency of the sucrose-best neuron to concentrations of sucrose in caterpillars exposed to one type of artificial diet in the F8 and F9 generation, and caterpillars exposed to other two artificial diets in F9 generation, *i.e.* caterpillars in ***F8-HS**, **F9-HS**, **F8-HS *****+***** F9-LS*** and ***F8-HS *****+***** F9-MS***. An one-way ANOVA followed by Post-hoc SNK test was used to compare: (1) the mean response frequency of the sucrose-best neuron in caterpillars with different feeding experiences in response to 1 mM sucrose; (2) the mean response frequency of the *myo*-inositol-sensitive neuron or the sinigrin-sensitive neuron in the medial sensillum of caterpillars from ***Wild**, **F1-HS**, **F9-HS**, **F9-LS*** in response to 1 mM *myo*-inositol or 1 mM sinigrin. The significance level was set at *P* < 0.05 or *P* < 0.01. All statistical analyses were conducted using SPSS version 10.0 (SPSS Inc., Chicago, IL, USA).

## Additional Information

**How to cite this article**: Ma, Y. *et al*. Trans-generational desensitization and within-generational resensitization of a sucrose-best neuron in the polyphagous herbivore *Helicoverpa armigera* (Lepidoptera: Noctuidae). *Sci. Rep.* **6**, 39358; doi: 10.1038/srep39358 (2016).

**Publisher’s note:** Springer Nature remains neutral with regard to jurisdictional claims in published maps and institutional affiliations.
